# Lipopolysaccharide (*LPS*) disrupts particle transport, cilia function and sperm motility in an *ex vivo* oviduct model

**DOI:** 10.1038/srep24583

**Published:** 2016-04-15

**Authors:** A. M. O’Doherty, M. Di Fenza, S. Kölle

**Affiliations:** 1 UCD School of Medicine and Medical Science, Health Sciences Centre, University College Dublin, Dublin 4, Ireland

## Abstract

The oviduct functions in the transportation of gametes to the site of fertilization (the ampulla) and is the site of early embryonic development. Alterations of this early developmental environment, such as the presence of sexually transmitted pathogens, may affect oviduct function leading to reduced fertilization rates and contribute to compromised embryonic development. In this study, sperm interactions, particle transport speed (PTS) and cilia beat frequency (CBF) in the ampulla following exposure to *lipopolysaccharide* (*LPS*), a constituent of the sexually transmitted pathogens *Chlamydia trachomatis* and *Chlamydia abortus*, was investigated. Three complementary experiments were performed to analyse; (1) bound sperm motility and cilia function (2) transport velocity in the oviduct and (3) the expression of genes related to immune function and inflammatory response (*CASP3*, *CD14*, *MYD88*, *TLR4* and *TRAF6*). The motility of bound sperm was significantly lower in ampullae that were exposed to *LPS*. CBF and PTS significantly increased after treatment with *LPS* for 2 hours. Finally, gene expression analysis revealed that *CASP3* and *CD14* were significantly upregulated and *TLR4* trended towards increased expression following treatment with *LPS*. These findings provide an insight on the impact of *LPS* on the oviduct sperm interaction, and have implications for both male and female fertility.

*Chlamydia trachomatis* (*C*. *trachomatis*) is the most frequently sexually transmitted human disease worldwide^1^. *C. trachomatis* can cause infection of the upper genital tract without any symptoms and can lead to damage of the oviduct (uterine tubes, Fallopian tubes), increasing the risk of future ectopic pregnancy and tubal infertility; consequently *C*. *trachomatis* is estimated to be the most costly nonviral sexually transmitted infection. In ruminants, *Chlamydia abortus* (*C. abortus*) is endemic worldwide and is a major causative agent associated with abortion and foetal loss in sheep and cattle^2^,^3^. In addition, a large proportion, 80–90%, of bovine uteri are contaminated with bacteria following parturition, which may persist leading to subclinical endometritis and reduced conception rates^4^,^5^,^6^. It is recognised that infection of the endometrium by pathogenic bacteria and viruses is preceded by endometrial infection with *Escherichia coli* (*E. coli*)^7^. This large scale postpartum infection has vast economic consequence, contributing to female reproductive disorders and the associated infertility which is estimated to cost €1.4 billion in the EU and $650 million per year in the United States^7^,^8^,^9^. In human, aberrant immune function in relation to infection is associated with adverse outcomes in many aspects of fecundity, such as ovarian function, embryo implantation and pregnancy loss^10^,^11^.

For successful fertilization sperm must first migrate through the cervix, transit through the uterus, enter the narrow passageway into the oviduct (the uterotubal junction) and swim against the tubal fluid flow before reaching the oocyte in the ampulla. The ampulla is the middle region of the three anatomical segments into which the oviduct is divided: the infundibulum (proximal to the ovary), ampulla and isthmus (proximal to the uterotubal junction and uterine horn). It has a central role in the early stages of development as it is the location where gametes undergo their final maturation, fertilization occurs and early embryonic development takes place^12^,^13^.

Over 20 years ago it was suggested that binding of bovine sperm to oviduct epithelial cells prolongs sperm motility in order to maintain fertilisation capacity^14^. These findings were confirmed by further studies showing that sperm are retained in the isthmus in a storage reservoir^15^, which is essential for preserving sperm fertility, as sperm remain bound until they are slowly released around the time of ovulation^16^. Additionally, it has also been demonstrated that sperm binding is not affected by the anatomical region of the oviduct or the cycle stage of heifers^17^. More recently, it has been demonstrated that *in vitro* incubation of human sperm with known pathogenic (*E. coli*), and conditionally pathogenic bacterial strains (Staphylococcus haemolyticus, Bacteroides ureolyticus) results in cellular death, which may be due to both apoptosis and necrosis^18^. The authors concluded that bacteriospermia may be a direct cause of subfertility or contribute to additional negative factors that may deteriorate the prognosis of fertility in natural and assisted reproduction. However, a previous study investigating the incidence of bacteriospermia and elevated seminal leukocytes in a cohort of subfertile Canadian males reported that, although prevalent in subfertile men, bacteriospermia was not statistically associated with subfertility^19^. Nevertheless, these studies highlight the importance for further interrogation of the relationship between infection and fertility.

Sperm viability is not the only condition for successful reproduction; the biophysical environment of the female genital tract, such as transport speed and cilia beat frequency, plays a crucial role to facilitate sperm migration into the oviduct and to block the access of sexually transmitted pathogens^20^. In spite of these defensive mechanisms, infection of the oviduct is still possible as epithelial cells in the oviduct are exposed to pathogens and endotoxins from the uterus and peritoneal cavity^21^,^22^.

Lipopolysaccharide (*LPS*) is a major constituent of the cell wall in most gram negative bacteria. Gram-negative bacteria (e.g. *C*. *trachomatis*, *C .abortus* and *E*. *coli*) signify a major class of pathogens that are thought to infect all eukaryotes^23^. Therefore, *LPS* is a useful molecule for mimicking bacterial infections, both *in vitro* and *ex vivo*. Stimulation of TLR4, a member of the Toll-like receptor family expressed on the cell surface and endosomes of monocytes, macrophages, dendritic cells, the oviduct and intestinal epithelium, with the bacterial endotoxin *LPS* induces intracellular signalling (involving TRAF6 and MYD88) that results in the release of critical proinflammatory cytokines, which are required for triggering effective immune responses^22^,^24^,^25^,^26^,^27^. Transcriptomic effects, following challenge with *LPS,* have previously been investigated *in vitro* using epithelial cells of the bovine oviduct and endometrium^5^,^28^. These studies demonstrated an upregulation of inflammatory response genes (e.g. *TRAF6*, and *CASP3*) and immune and stress response genes (*TLR4*, *MYD88* and *CD14*). However the response of the oviduct to *LPS* exposure, *ex vivo*, remains unknown.

The objective of the current investigation was to develop an *ex vivo* model of infection for studying sperm interactions in the oviduct. To this end, we developed a method using explants of the bovine oviduct and *LPS* to elucidate the physiological and molecular changes elicited by *LPS* on oviduct function and sperm-oviduct interactions. In respect of that, we combined previously established video microscopy imaging techniques^29^ with gene expression analysis to determine the effect of an *LPS* challenge on (1) bound sperm motility, (2) particle transport speed, (3) cilia beat frequency and (4) abundance of inflammatory, immune and stress response genes.

## Results

### Exposure of ampulla to LPS alters sperm motility

**Figure 1:**
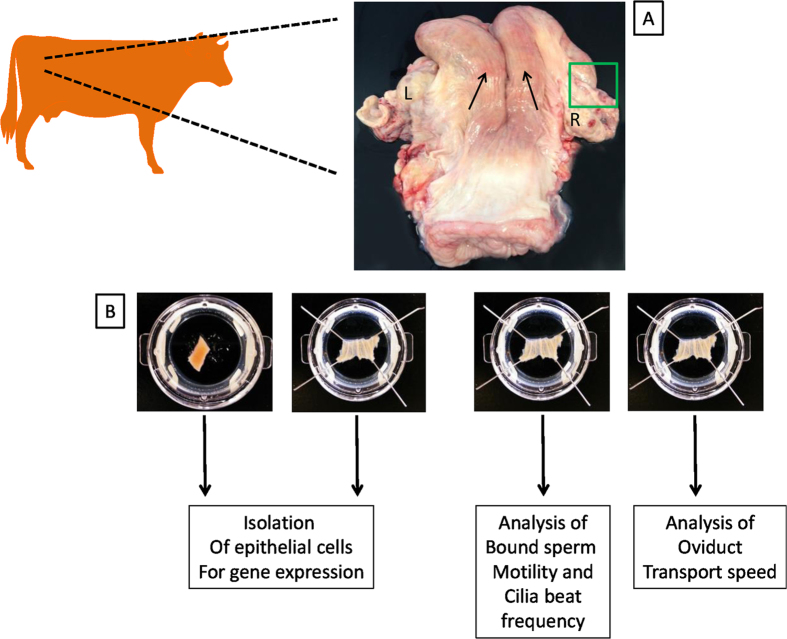
Schematic representation of the experimental method. Oviducts were isolated from slaughterhouse reproductive tracts (**A**) that were determined to be in the diestrus stage of the bovine reproductive cycle. Sections of the ampulla (**B**) were pinned or not pinned according to experiment being carried out. L = left ovary and R = right ovary. Black arrows show the location of the left and right uterine horns and the green box highlights the position of the right oviduct. With the exception of the cow schematic, this figure was drawn by AOD using photographs captured by MDF. The cow was adapted from the PPT Drawing Toolkits BIOLOGY Bundle, Motifolio // www.motifolio.com/.

**Figure 2:**
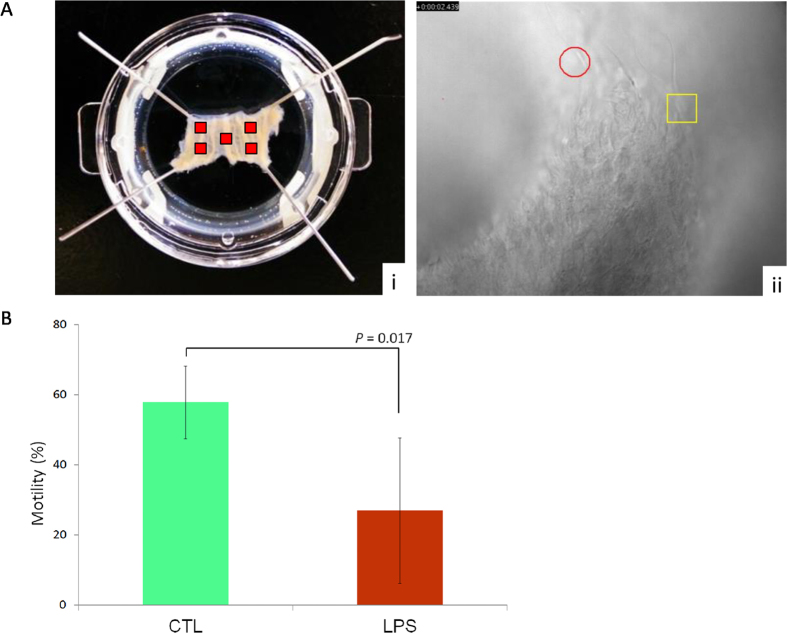
Motility of bound sperm in oviduct challenged with *LPS* compared to control. Video microscopy was used to capture spermatozoa bound to the epithelium in sections of the bovine ampulla. (**A**) The motility of bound sperm was determined in 5 regions (represented by red boxes in (i)) using ImagePro software, bound sperm that were motile were labelled with a square and bound sperm that were immotile with a circle (ii). (**B**) The number of motile sperm (represented as a percentage of the total number of bound sperm counted) was significantly reduced following exposure to *LPS* (*P* < 0.05).

In the first experiment, uncapacitated sperm that were bound to sections of ampulla, which had been pre-incubated with 10 μg/ml *LPS*, were assessed for motility (Fig. 1). After addition of frozen thawed spermatozoa of bulls with proven fertility, it was observed that sperm rapidly attached to ciliated epithelial cells of the ampulla sections. Subsequently, the buffer was replaced after 30 and 60 min to remove unbound sperm and to ensure that sperm being captured at 90 min were bound. The motility of sperm, which were bound to the ampulla, was recorded in 5 defined regions of each video captured (Fig. 2Ai). Sperm were scored as either being motile or immotile and percentage motility was determined by comparing the ratio of bound motile to bound immotile sperm (Fig. 2Aii). The total number of individual bound sperm assessed under control and *LPS* challenge conditions were, CTL = 249 and *LPS* = 157. Although it appeared that less sperm were bound following exposure of the ampulla to *LPS* the difference was not statistically significant (*P* = 0.16). However, analysis of bound sperm motility revealed that it was significantly compromised in the *LPS* group (*P* < 0.05) with an average motility of 27%, about half the motility (58%) of bound sperm in the control group (Fig. 2B). Representative videos of sperm motility with and without *LPS*-challenge are located in the supplementary information (Supplementary videos 1 and 2).

### Increased particle transport speed following LPS challenge

**Figure 3:**
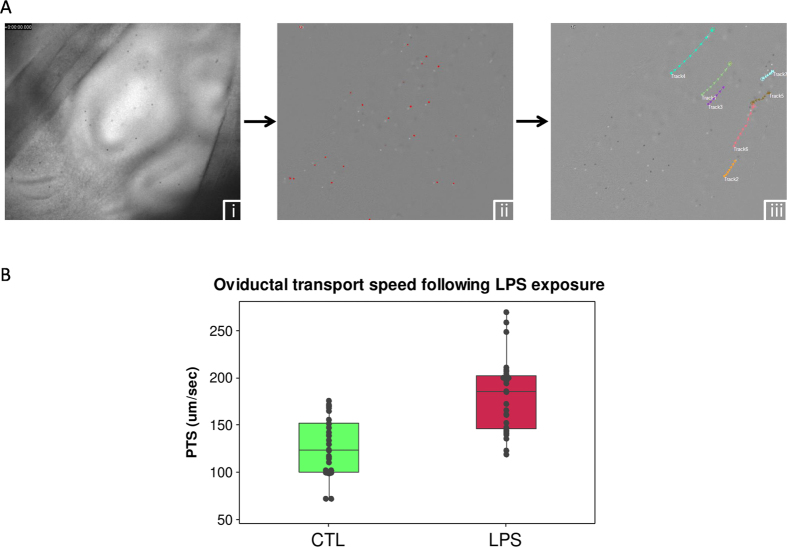
Influence of *LPS* on particle transport speed in the oviduct. (**A**) Representative images of the method used to determine particle transport speed. (i) Raw image showing the presence of the particles (2.8 μM Dynabeads) in the oviduct. (ii) Removal of background and labelling of particles (red) Using ImagePro. (iii) Tracking of particles. (**B**) Particle transport speed (PTS) was significantly increased in ampulla sections that had been incubated in the presence of LPS for 2 h (*P* < 0.05). Data are presented as the mean ± standard deviation. Closed black circles represent the average PTS for each individual video used in the analysis (Total number of oviducts analysed, *n* = 5, Total number of PTS measurements, CTL *n* = 30 and *LPS n* = 29).

Transit of gametes and early embryos through the oviduct is likely to be affected by the tubal fluid flow, which is managed by a) ciliary activity and b) smooth muscle contraction. In order to gain an insight into the physiological impact of *LPS* on oviduct function, transport speed was investigated in a second series of experiments (Figs 1 and 3A). Sections of ampulla that were exposed to 10 ug/ml *LPS* (*n* = 5) for 2 h were compared to control sections that were maintained in HEPES buffer (CTL *n* = 5). Six videos were recorded for all samples giving a total number of 30 for both CTL and *LPS* (Supplementary videos 3 and 4). After this relatively short exposure to *LPS*, the transport speed was significantly increased in *LPS*-exposed oviducts, CTL = 127 ± 5.5 vs *LPS* = 181.8 ± 7.2 μm/sec (mean ± SEM) (*P* ≤ 0.001) (Fig. 3B). A set of complementary PTS experiments were carried out, using ampulla sections from tracts similar to those described in supplementary file 1, to elucidate the directionality of tubal flow in the bovine oviduct (3 AOIs from two biological replicates, supplementary videos 5 and 6). These experiments demonstrated that particles transit in the direction of the uterus. Further investigation of videos recorded for PTS analysis revealed that the flow was in the same direction for each AOI in each replicate. Taken together, the direction of particle transport observed in both experiments suggests that tubal flow is unidirectional in these samples and is in accordance with what has previously been reported for orientation of tubal flow during diestrus, i.e. in the direction of the uterus^30^,^31^.

### Does LPS affect cilia function?

**Figure 4:**
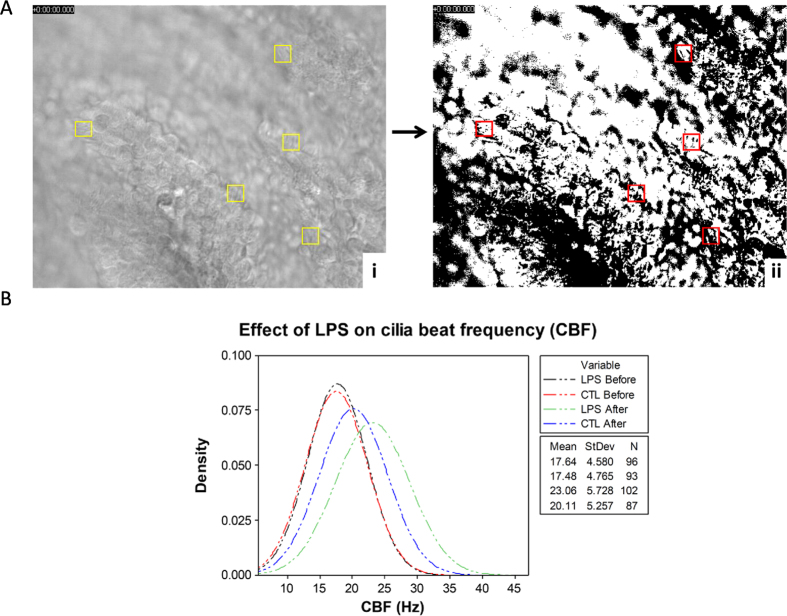
Cilia function in response to *LPS* exposure. Cilia beat frequency (CBF) was analysed in the ampulla before and after exposure to *LPS* and compared to the CBF of ampulla sections that were unexposed (CTL) using ImageJ software. (**A**)(i) Videos were captured at 100 frames per second and single ciliated cells were selected (yellow boxes) as areas of interest (AOI) before a binary mask was applied using the convert to binary function (ii). The AOIs are represented by red boxes following masking. (**B**) No significant differences in CBF were observed in CBF before *LPS* treatment (*P* > 0.05). CBF was significantly higher in the LPS group after incubation with *LPS* for 2 h (*P* > 0.05). CTL and *LPS* before videos *n* = 25 each. CTL and *LPS* after videos *n* = 25 each. Total number of AOIs analysed; CTL-before *n* = 100, *LPS*-before *n* = 100, CTL-after *n* = 100 and *LPS*-after *n* = 100.

Subsequent to identifying that transport speed was significantly increased after treatment with *LPS* we hypothesised that increased PTS occurs as a result of elevated cilia beat frequency. To test this hypothesis we recorded videos in oviducts that were either (1) maintained in HEPES buffer (CTL) or (2) exposed to *LPS*. CBF was analysed for both groups, before and after 2 h incubation at 37 °C (Fig. 1). Videos were captured using the same method described for analysis of sperm (Fig. 2Ai). The method used to determine CBF is outlined in Fig. 4A (See supplementary videos 7 and 8 for representative videos). As expected, no significant differences were observed for CBF between the CTL and *LPS* samples at the beginning of the experiment prior to incubation (CTL-before = 17.5 ± 4.8 Hz vs *LPS*-before = 17.6 ± 4.6 Hz, *P* ≥ 0.05) (Fig. 4B). However, samples that were incubated in *LPS* for 2 h showed a significant increase in CBF, relative to those that were unexposed CTL-after = 20.1 ± 5.2 Hz vs *LPS*-after = 23.1 ± 5.1 Hz, *P* ≤ 0.05) (Fig. 4B). These observations are in agreement with what was observed for particle transport in response to *LPS* exposure. Additionally, in this model there appeared to be an increase in CBF frequency over time in both control and *LPS*-treated samples (*P* ≤ 0.05).

### Effect of *ex vivo* LPS exposure on expression of inflammatory and immunological response genes

**Figure 5:**
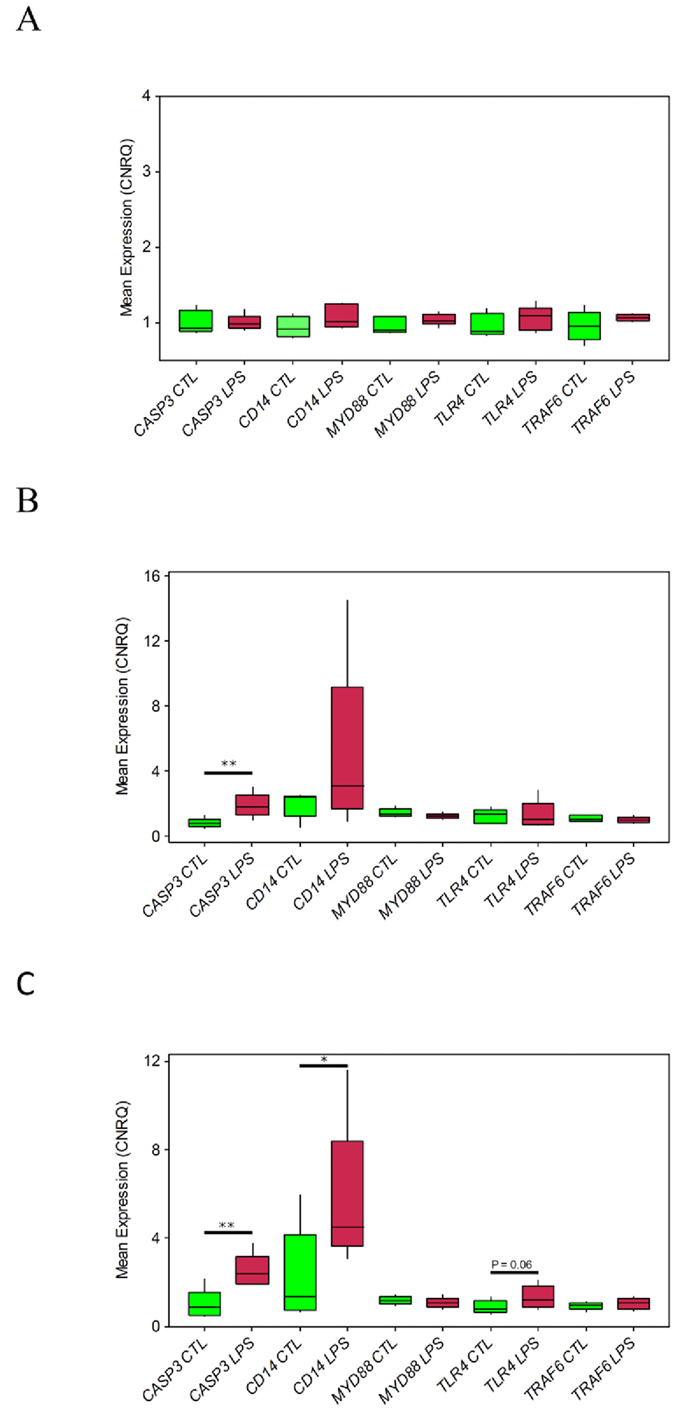
Expression patterns of inflammatory and immunological response genes after *LPS* challenge. Calibrated normalised relative quantities (CNRQ) were plotted using the boxplot function in Mintab v16. Whiskers and boxes represent outliers and the interquartile range, respectively. No significant differences (*P* > 0.05) in expression were observed for any of the genes in epithelial cells isolated from intact sections of oviduct (**A**). Only *CASP3* was significantly upregulated (*P* < 0.05) in epithelial cells isolated from longitudinally dissected pieces of oviduct exposed to 10 μg/ml *LPS* (**B**) however both *CASP3* and *CD14* were significantly upregulated when the concentration was increased to 100 μg/ml (**C**).

Gene expression analysis was performed to determine if *LPS* was exerting an effect on the epithelial cells of *ex vivo* sections of ampulla, relative to untreated control samples (Fig. 1). The gene expression profiles of a set of five target genes (*CASP3*, *CD14*, *MYD88*, *TLR4* and *TRAF6*), which have been shown to be up regulated in inflammatory, immunological and stress responses *in vitro*^28^, were characterised using three separate, but complementary, methods. No significant changes (*P* > 0.05) in the expression of any of the target genes was observed in epithelial cells isolated from closed sections of oviduct that were exposed to 10 μg/ml of *LPS* for a period of 2 h (Fig. 5A). Therefore, expression of the target genes was assessed in a second set of samples in which the section of ampulla had been dissected longitudinally through the lumen prior to *LPS* exposure. In this experiment the expression of *CASP3* was significantly higher (*P* < 0.05) in ampulla that was challenged with *LPS* group (Fig. 5B). In a final set of experiments the concentration of *LPS* was increased from 10 μg/ml to 100 μg/ml and the incubation time increased to 3 h. Again, *CASP3* expression was significantly higher (*P* < 0.05) in the ampulla sections exposed to *LPS* (Fig. 5C). Additionally, *CD14* expression was also higher (*P* < 0.05) in the *LPS* challenged sections and *TLR4* had trended towards increased expression (Fig. 5C).

## Discussion

The effects of clinical and sub-clinical endometritis on fertility have been extensively studied using the bovine model^5^. However, limited information is known about the impact of infection in the bovine oviduct on fertility. The current investigations provide novel information on the impact of *LPS*, a key constituent of *E. coli* and the sexually transmitted pathogens *C. trachomatis* and *C. abortus*, on bound sperm motility, transport speed, cilia function and inflammatory and immunological gene expression patterns in an *ex vivo* model of oviduct infection.

The elevated speed and frequency observed in particle transport and cilia beating, respectively, under *LPS* challenge may represent a host response mechanism to increase fluid flow during the early stages of infection, in which the oviduct is attempting to remain sterile by flushing foreign particles towards the uterus^20^. This hypothesis is supported by previous evidence demonstrating that the fluid flow is unidirectional^30^,^31^, and that it represents a defensive mechanism against the invasion of pathogens^13^. Indeed, elevated CBF during infection is not uncommon as it has been demonstrated that cilia beat frequency is increased in murine and human *ex vivo* mucus-free airway preparations, when challenged with *LPS*^32^. In the current study, particles were shown to move in the direction of the uterus, and *LPS* was shown to increase both CBF and PTS. Although *LPS* clearly has an impact on the oviduct through increasing cilia beating and transport speed, the consequences of *LPS* on sperm transit through these conditions, *in vivo*, remains less clear. Indeed, it has been demonstrated that sperm are capable of swimming against the oviductal fluid flow (a process known as positive rheotaxis)^33^. Additionally, using microfluidic modelling it has been shown that microgrooves and gentle fluid flows provide preferential pathways that facilitate sperm transport against fluid flow^20^. Therefore, it is possible that sperm are capable of transiting against the increased fluid flow in oviducts that have been exposed to *LPS*, however to what extent they can go against the flow remains undetermined. Irrespective of the impact of *LPS* on sperm transport through the oviduct, results of this study show that the motility of bound sperm is significantly reduced.

It was recently demonstrated that exposure of bovine oviduct epithelial cells to *LPS* resulted in elevated expression of inflammatory and immunological response genes within three hours *in vitro*^28^. The authors identified an immediate response of oviduct epithelial cells to *LPS* through upregulated expression of *TLR4* and associated genes (*MYD88* and *CD14*) downstream in its pathway. This demonstrates that canonical *LPS*-*TLR4* mediated signalling is occurring as expected^34^,^35^,^36^. Expression patterns of a selected panel of inflammatory and immunological response genes were assessed in the current study to determine if a similar response occurred in our *ex vivo* model. Although expression of *CD14* increased, no increase in *TLR4* expression was observed following challenge of the oviduct with *LPS*, suggesting that in this model *LPS* is not stimulating the canonical *TLR4* signalling pathway and that, instead, a *TLR4*-independent mechanism may be in place. *TLR4*-independent pathways have been described by several groups and suggest that *LPS* stimulates non-canonical inflammasome activation in a caspase-1/caspase-11 dependent manner in mice^35^,^37^,^38^; whereby caspase-11 binds directly to *LPS* resulting in caspase oligomerization and inflammasome activation^39^. This non-canonical inflammasome activation has also been observed in human, where caspase-4 is involved with mediating a noncanonical response against gram-negative bacterial pathogens^40^.

Additionally, it was demonstrated that the expression of *CASP3*, a biological indicator and known executioner of apoptosis^41^,^42^,^43^, was upregulated following exposure of the oviduct to *LPS* in the current study. Increased *CASP3* activity could be indicative of an apoptotic response by the bovine oviduct, mediated through *LPS*. It has, however, been shown using *in vivo* and *in vitro* models of microglia activation and brain inflammation that *LPS*-mediated induction of caspases-3, -8, and -7 results in a non-apoptotic response, as major cell death was not observed^44^. The authors of this investigation concluded that these caspases have a pivotal role in inflammation of the central nervous system. Therefore, *LPS*-induced activation of *CASP3* in the ampulla observed in this study does not necessarily indicate an apoptotic response to *LPS*, and may be indicative of a similar inflammatory response described in^44^.

Classically, the oviduct is defined as a sterile environment, however pathogens and endotoxins (*LPS*) can invade the mucosal surfaces of the oviduct via the uterus and peritoneal cavity^22^. It has also been shown that, in cattle, *LPS* is detectable in ovarian follicular fluid; isolated *in vivo* from animals with different severities of uterine disease^21^. In this study, the authors showed that animals with clinical disease had concentrations of *LPS* up to 0.8 ug/ml and normal animals did not have measurable concentrations of *LPS*. This provides evidence that animals suffering uterine infection contain *LPS* in their follicular fluid, which may be delivered directly into the oviduct following ovulation. However, whether sperm can transit through infected uteri of animals with uterine disease remains largely unknown, therefore *LPS* or bacteria associated with uterine infection may elicit an effect on sperm prior to their transition into the oviduct. Females suffering *C. trachomatis* or *C. abortus* infections are often asymptomatic and subfertile, as opposed to completely infertile^1^,^45^. It is very plausible that in these females sperm encounter oviducts that contain or have been exposed to *LPS*. Therefore, the current study best represents a potential environment in females suffering Chlamydia infections. It has been previously suggested that bacterial endotoxins (*LPS*) can act directly on sperm through activation of TLRs present on their membranes, resulting in reduced sperm motility, apoptosis and, possibly, impairment of fertilization potential^46^. Results of the current study are in direct agreement with the observation of Fujita *et al.*^46^, given that bound sperm motility was reduced by 50% following a short exposure to *LPS*. Therefore, *LPS* may be exerting and effect on not only the oviduct, but may be directly affecting the sperms ability to interact with the oviductal epithelium and may also be involved with reducing the motility of the sperm that are capable of interacting with the epithelium. The impact of the observed reduction in sperm motility may disrupt its ability to interact with the oocyte, once present in the ampulla. However, at the current time this is only a hypothesis and future research should focus on elucidating the significance of reduced sperm motility following exposure to *LPS*.

In summary, we provide the first insight into sperm behaviour, in real time, in *ex vivo* oviduct sections following exposure to *LPS*. Oviduct function was significantly altered at both the physiological and the genetic level subsequent to a relatively short exposure to the gram negative bacterial endotoxin, *LPS*. These results demonstrate a rapid host response of healthy oviducts to *LPS*, through elevated tubal flow and increased frequency of cilia movement. The motility of sperm interacting with the oviduct was significantly affected by exposure to *LPS* and suggests that even a short exposure of the oviduct to infection may have dire consequences on fertility. Finally, the findings of this investigation provide evidence demonstrating that the cow is an appropriate model for studying reproductive diseases and immunity.

## Materials and Methods

### Experimental Overview

The physiological, inflammatory and immunological response of the bovine oviduct to *LPS* treatment was investigated. Firstly, bound sperm motility, PTS and CBF were interrogated using live cell imaging microscopy to examine the physiological response of ampulla to *LPS* treatment, Secondly, to investigate the inflammatory and immunological response of the oviduct epithelial cells to *LPS*, genes known to respond to *LPS* exposure *in vitro* (*CASP3*, *CD14*, *MYD88*, *TLR4* and *TRAF6*)^28^ were analysed by real time PCR. Experiments were performed as outlined in Fig. 1.

### Sample preparation and LPS treatment

Female reproductive tracts from 5 separate recently slaughtered animals were collected at a local abattoir, transported to the laboratory on ice within an hour and determined to be in the diestrus stage of the oestrus cycle based on (1) presence of a large corpus luteum on one of the ovaries (2) closed cervix (3) absence of mucus in the uterine horn and cervix. Details of the tracts used in this study (Sperm binding, PTS, CBF and the first gene expression experiment) are outlined in supplementary file 1. Ten additional tracts were collected for qPCR experiments (Fig. 5B,C). Identical parameters were used to select oviducts for the second and third qPCR experiments.

Tracts were prevented from drying out with PBS while the ipsilateral oviduct was being dissected from the uterus, trimmed free from connective tissue and each ampulla was divided in four 5 mm and two 10 mm segments. Two 5 mm segments, one for control conditions and the other for challenge with *LPS*, were used to measure cilia beat frequency (CBF) and bound sperm motility; the other two 5 mm to measure particle transport speed (PTS) with and without exposure to *LPS*. The 10 mm segments, one maintained in HEPES and the other in HEPES + *LPS*, were used to isolate the RNA for gene expression profiling.

The four 5 mm segments were washed in PBS, opened longitudinally and pinned down, with 1 cm pins recovered from 26 G Sterican needles (Braun, GmbH, Germany), on Delta T dishes (Bioptechs, PA, USA) coated with a 1 mm Sylgard 184 (Dow Corning, MI, USA) layer. The two 10 mm segments were transferred in a four-well dish filled with 1 ml PBS. Pinned sections were resuspended in 1.5 ml PBS a couple of times until cell debris was fully removed and discarded, then they were submerged in 1.5 ml room temperature HEPES buffer solution (5.6 mM KCl, 136.4 mM NaCl, 1 mM MgCl_2_-6H_2_O, 2.2 mM CaCl_2_-2H_2_O, 11 mM glucose, 10 mM HEPES) with and without 10 μg/ml *LPS* (Cat. No. L6529, Sigma-Aldrich, Germany). Ampulla segments in the four-well dish were gently washed by flushing PBS through the lumen and were finally submerged in 1 ml lukewarm HEPES buffer solution with and without 10 μg/ml *LPS*. The concentration was selected based on previous studies. Ibrahim *et al.*^28^ used a minimal dose of 0.5 μg/ml *LPS*. Given that these conditions were used in an *in vitro* cell culture model (in which monolayers of cells are homogenously exposed to *LPS*) and that 0.5 μg/ml was described as a minimal dose, a concentration of 10 μg/ml was used in the current *ex vivo* model. This concentration has also been used in other *in vivo* and *in vitro* models of infection^21^,^47^,^48^.

### PTS analysis

Two ampulla sections from the ipsilateral oviduct of each animal (*n* = 5) were incubated in Petri dishes as per CBF for 2 h in HEPES buffer solution at 37 °C. After incubation, the dishes were transferred to the Delta T stage adapter and maintained at 37 °C. 3 μl (equivalent to 90 μg) of 2.8 μm Dynabeads Protein G (Life Technologies, AS, Norway) were added to the dish and gently resuspended a few times. Videos were recorded, in duplicate, on three different regions at 12 frames per second (FPS) for 10 sec, 640 × 480 pixel resolution. Videos were converted to 8-bit grayscale and particles were tracked with an automatic tracking procedure in-built in ImagePro (MediaCybernetics, PA, USA). Only the particles that travelled for at least 10 frames were used for calculation.

### CBF analysis

Two sections of ampulla from the oviducts (ipsilateral side) of each animal (*n* = 5) were equilibrated in HEPES buffer solution at 37 °C in incubator for 10 min. To reduce evaporation, Delta T dishes were transferred in two humidity chambers consisting in two 10 cm closed Petri dishes whose bottom was coated with two layers of moist filter paper. Following equilibration, dishes were transferred to a Delta T stage adapter (Bioptechs, PA, USA) where temperature was controlled and maintained at 37 °C. Ciliary beating was assessed with a pre-warmed 40 × W/0.70 water immersion objective (Olympus, Hamburg, Germany) maintained at 37 °C by an objective heater (Bioptechs, PA, USA). Images were captured with the StreamPix 7.0 (NorPix, Canada) software connected to a SUMIX Mx7 camera (100 FPS/10 sec, 640 × 480 pixel resolution) mounted on a BX51WI fixed-stage upright microscope (Olympus, Hamburg, Germany). Videos were recorded on five different areas of interest (AOI) per sample (Fig. 2Ai) the central area and the four corners of each section. After imaging, dishes were emptied and replenished with a suspension of 5 × 10^6^ spermatozoa in 1.5 ml HEPES buffer solution for the control and with 1.5 ml HEPES buffer solution supplemented with *LPS* for the treatment. The dishes were then incubated at 37 °C for 2 h during which videos were recorded every 30 min as previously described and buffer solutions were swapped with fresh ones before transferring the dishes back in the incubator.

Cilia beat was measured on 5 ciliated cells per AOI. The videos were converted to black and white by applying a binary mask using ImageJ (National Institutes of Health). Ciliated cells were selected within regions of approximately 40 × 40 μm, and 400 frames were analysed. Average values and frames were exported to AutoSignal (Systat Software GmbH) and frequency was calculated (Hz) using Fast Fourier Transformation (FFT).

### RNA extraction and cDNA synthesis

Oviduct sections were subjected to three separate methods of exposure to *LPS* prior to harvesting for RNA extraction; all incubations were carried out at 37.5 °C. Firstly, oviduct epithelial cells were isolated from intact longitudinal 1 cm sections of ampulla, which had been challenged with 10 μg/ml *LPS* for 2 h, using non-serrated forceps in a stripping motion. Secondly, 1 cm sections of ampulla were dissected longitudinally prior to being challenged with 10 μg/ml *LPS* for 2 h. Cells were recovered with non-serrated forceps as above. Finally, longitudinally dissected sections of ampulla were challenged with 100 μg/ml *LPS* for 3 h. Epithelial cells were harvested by gently scrapping the sections with a glass slide. The buffers were changed after 1 h (1.5 h for the final method) and cells were immediately snap frozen and stored at −80 °C. All experiments had five independent biological replicates of oviduct sections exposed to *LPS* and control sections that were not exposed to *LPS*. Total RNA was extracted using an RNeasy micro kit (Qiagen) as outlined previously^49^. cDNA was synthesised from 200 ng of total RNA in 20 μl reactions, using the high-capacity cDNA reverse transcription kit (Applied Biosystems Inc), as follows; 1 × RT buffer, 5 μm random primers, 4 mM dNTPs, 40 U RNase inhibitor and 50 U Multiscribe RT. Reverse transcription reactions were incubated at 25 °C for 10 min, 37 °C for 120 min and 85 °C for 5 min.

### Real Time PCR

Information on the genes analysed in this study has been published previously^50^ and the sequences of the primers that were used are outlined in Table 1. The expression of five candidate genes (*CASP3*, *CD14*, *MYD88*, *TLR4* and *TRAF6*) was examined in all control and *LPS*-exposed oviduct samples. The geNorm function in qBase^PLUS^ (Biogazelle, Zwijnaarde, Belgium)^51^ was used to analyse the stability of the candidate reference genes across all samples. The most stable reference genes, *RPL19* and *PPIA*, were used for normalization. Real Time PCR was performed using the 7500 Fast Real-Time PCR system (Applied Biosystems^®^, USA).

### Preparation of sperm

Sperm straws (Cryopreserved in BioxCell extender) from the same bull sire were thawed at 39 °C for 10 sec, resuspended 1 ml HEPES buffer solution and washed at 200 rcf/5 min. Supernatant was discarded and pellet was resuspended in 100 μl non-capacitating HEPES buffer solution (5.6 mM KCl, 136.4 mM NaCl, 1 mM MgCl_2_-6H_2_O, 2.2 mM CaCl_2_-2H_2_O, 11 mM Glucose, 10 mM HEPES). Sperm motility was visually assessed before and after wash using a standard phase contrast microscope. Only sperm with motility ≥70% were used for experiments. Spermatozoa were then quantified using a Neubauer chamber prior to addition to oviduct sections. Uncapacitated sperm were used in the experiments, as capacitation has been shown to reduce sperm binding to oviduct epithelial cells^52^,^53^.

### Analysis of Bound Sperm Motility

Following an initial incubation of the ampulla for 2 h, in either HEPES buffer or *LPS*, 5 × 10^6^ sperm were added to each section of ampulla. Five AOIs per sample were captured (*n* = 5 biological replicates) 90 min following addition of the sperm to the oviduct. Preliminary examination of the ampulla was performed to confirm that spermatozoa were interacting with the surface of the ampulla. The total number of motile and immotile sperm in each AOI was documented using Image Pro (MediaCybernetics). Motility was calculated by determining the ratio of motile to immotile bound sperm and is represented as a percentage.

### Statistics Analysis

Bound sperm motility and PTS analysis were performed using a two sample *t*-test and CBF analysis was performed using an ANOVA test with ad-hoc tukeys in Mintab (Minitab Inc.). Statistical analysis of gene expression was performed using the calibrated normalised relative quantities, based on normalisation factors generated for the two reference genes (*PPIA* and *RPL19*), for each target gene using the Mann-Whitney function in the qbase + package (Biogazelle).

## Additional Information

**How to cite this article**: O’Doherty, A. M. *et al.* Lipopolysaccharide (*LPS*) disrupts particle transport, cilia function and sperm motility in an *ex vivo* oviduct model. *Sci. Rep.* **6**, 24583; doi: 10.1038/srep24583 (2016).

## Supplementary Material

Supplementary Video 1

Supplementary Video 2

Supplementary Video 3

Supplementary Video 4

Supplementary Video 5

Supplementary Video 6

Supplementary Video 7

Supplementary Video 8

Supplementary File 1

## Figures and Tables

**Table 1:** Primer sequences for qPCR.

Gene	Forward Primer Sequence 5′–3′	Reverse Primer Sequence 5′–3′	RefSeq Accession Number
*CASP3*	GTC TGA CTG GAA AAC CCA AAC TTT	CTG TCT CAA TAC CAC AGT CCA GTT CT	NM_001077840.1
*CD14*	ACA GTC CAG CCG ACA ACC A	GGC ACG CAC ACC ATA GTC AGT	NM_174008.1
*MYD88*	CGA CGA CGT GCT GCT GAT GGA	CTC CTG CTG CTG CTT CAG AA	NM_001014382.2
*PPIA*	CAT ACA GGT CCT GGC ATC TTG TCC	CAC GTG CTT GCC ATC CAA CC	NM_178320.2
*RPL19*	GAA AGG CAG GCA TAT GGG TA	TCA TCC TCC TCA TCC AGG TT	NM_001040516.1
*TLR4*	CTG CCT GAG AAC CGA GAG TTG	GCT CCA TGC ACT GGT AAC TAA TGT	NM_174198.6
*TRAF6*	AGG CAT CTT GAG GAG CAT CAA	GGG ACG CTG GCA TTG C	NM_001034661.2
