# Supplementary material for: Lipopolysaccharide (LPS) disrupts particle transport, cilia function and sperm motility in an ex vivo oviduct model
Source: Sci Rep. 2016 Apr 15;6:24583. doi: 10.1038/srep24583 (PMC4832340; doi:10.1038/srep24583)
Supplement: Supplementary File 1 [file srep24583-s9.pdf]

Lipopolysaccharide (*LPS*) disrupts particle transport, cilia function and sperm motility in an *ex vivo* oviduct model

O'Doherty, A.M. [1, 2], Di Fenza, M.[1] and Kölle, S.[1]

[1] UCD School of Medicine and Medical Science, Health Sciences Centre, University College Dublin, Dublin 4, Ireland.

Supplementary Information File

Tract ID *LPS* 1 21-05-15

Left	Right (used experiment)
Ovary:<br>CL = few old<br>Follicles = few small, two medium<br>Size L/W/H (cm) = 3/2/1.5	Ovary:<br>CL = one large, red/orange<br>Follicles = several medium, very few small<br>Size L/W/H (cm) = 3.5/2.5/2
Horn:<br>Mucus = no (-)<br>Caruncles = yes<br>Oedema = no<br>Colour = pink/normal	Horn:<br>Mucus = no (-)<br>Caruncles = yes<br>Oedema = no<br>Colour = pink/normal
Oviduct:<br>vascularization = normal<br>Colour = pink	Oviduct:<br>vascularization = normal<br>Colour = pink

Cervix:
Mucus = no
Open = no

Straw information:
- *National Cattle Breeding centre*
- Storage solution = white
- Straw ID = LEANAMORE OMAN IE191038420409

ID:LPS 1
21-05-15

LEFT

RIGHT

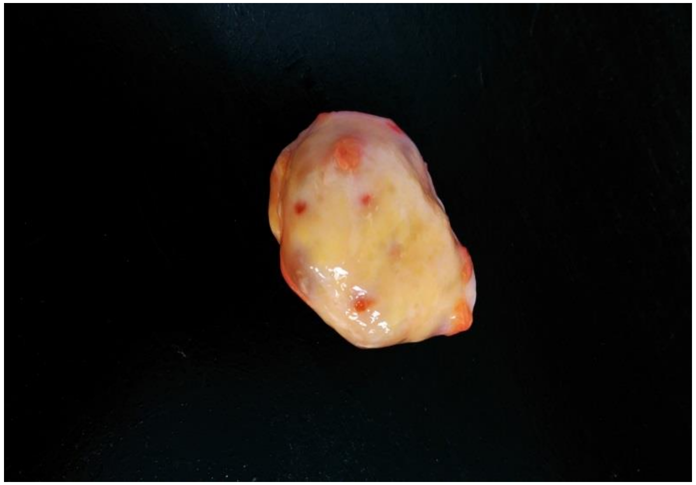

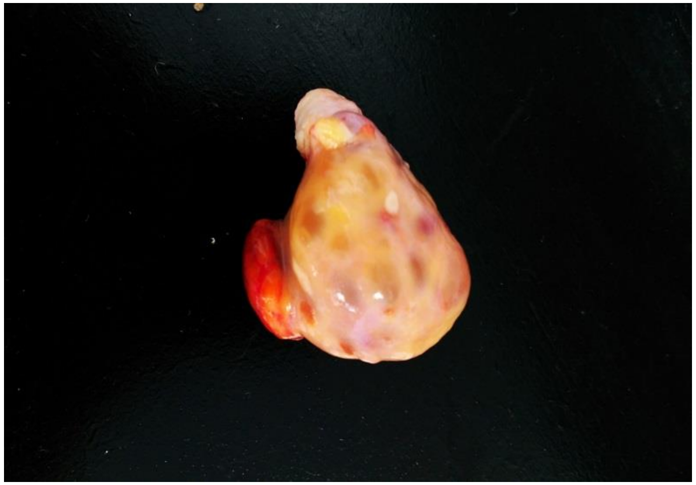

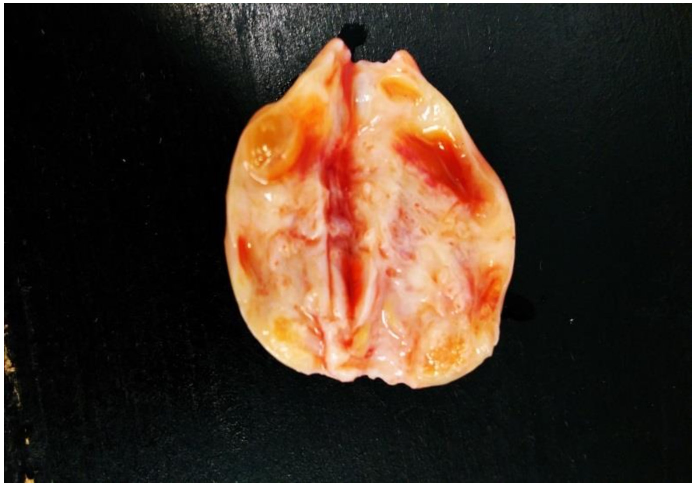

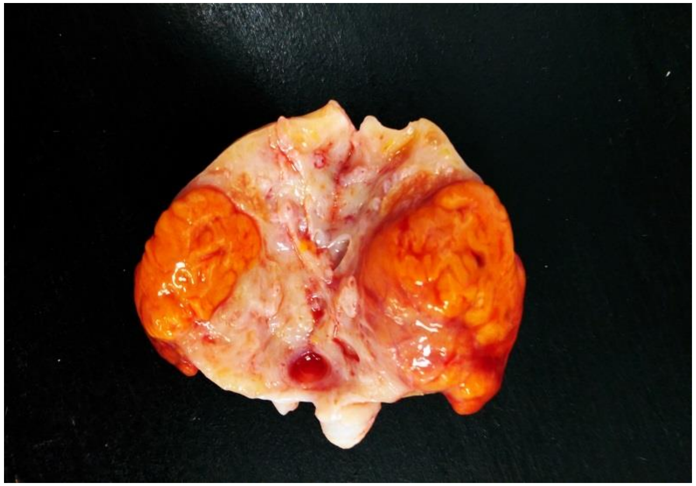

Tract ID *LPS* 2
29-05-15

Left (used for experiment)	Right
Ovary:<br>CL = One large orange and one old visible<br>Follicles = a few small<br>Size L/W/H (cm) = 3.1/2.8/2	Ovary:<br>CL = a few old visible<br>Follicles = one medium (~1cm) and a few small<br>Size L/W/H (cm) = 4/2.3/2.5
Horn:<br>Mucus = no (-)<br>Caruncles = yes<br>Oedema = no<br>Colour = pink/normal	Horn:<br>Mucus = no<br>Caruncles = yes<br>Oedema = no<br>Colour = pink/normal
Oviduct:<br>vascularization = normal<br>Colour = pink	Oviduct:<br>vascularization = normal<br>Colour = pink

Cervix:
Mucus = no
Open = no

Straw information:
- *National Cattle Breeding centre*
- Storage solution = white
- Straw ID = LEANAMORE OMAN IE191038420409

ID:LPS 2
29-05-15

LEFT

RIGHT

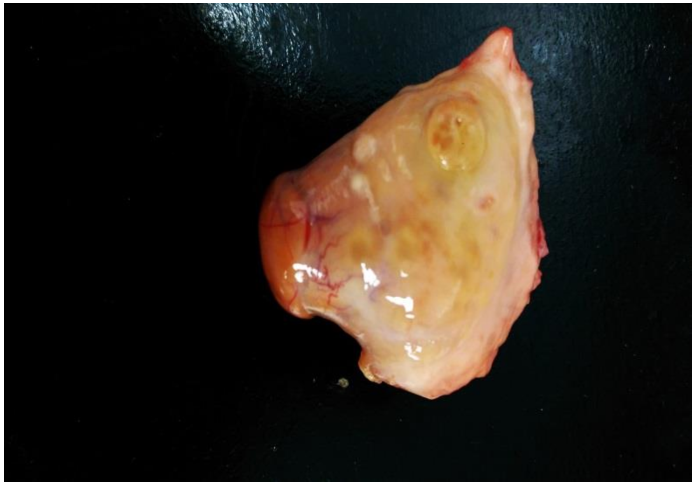

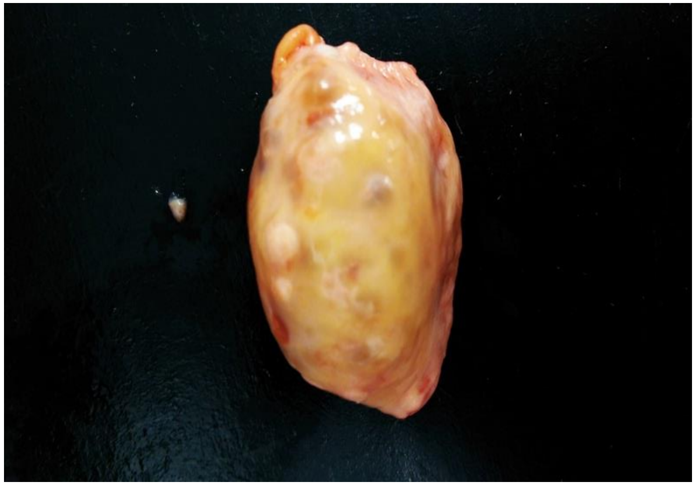

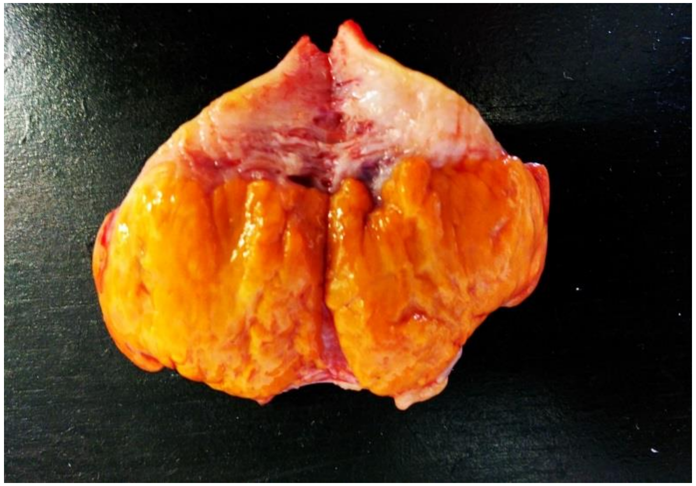

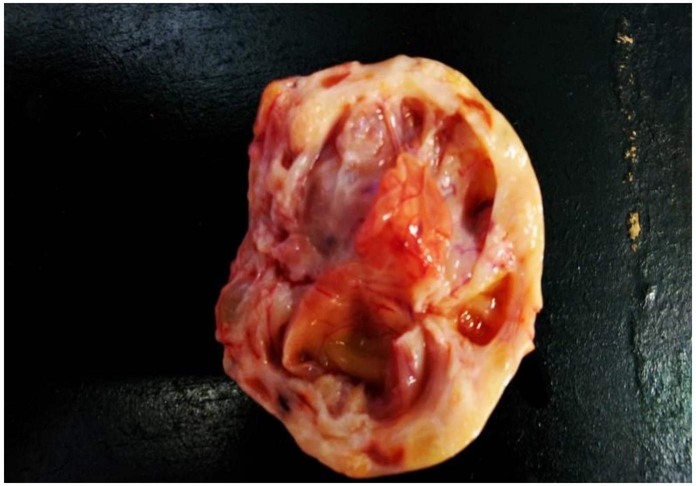

Tract ID *LPS* 3
02-06-15

**Left (used for experiment)**	**Right**
Ovary:<br>CL = One large orange and a few old visible<br>Follicles = one large (1.4cm), a few medium and several small<br>Size L/W/H (cm) = 4.5/3/2	Ovary:<br>CL = few old, white CL's visible<br>Follicles = one medium (~1cm) and several small<br>Size L/W/H (cm) = 3.3/2/2
Horn:<br>Mucus = no<br>Caruncles = yes<br>Oedema = no<br>Colour = pink/normal	Horn:<br>Mucus = no<br>Caruncles = yes<br>Oedema = no<br>Colour = pink/normal
Oviduct:<br>vascularization = normal<br>Colour = pink	Oviduct:<br>vascularization = normal<br>Colour = pink

Cervix:
Mucus = no
Open = no

Straw information:
- *National Cattle Breeding centre*
- Storage solution = white
- Straw ID = LEANAMORE OMAN IE191038420409

ID:LPS 3
02-06-15

LEFT

RIGHT

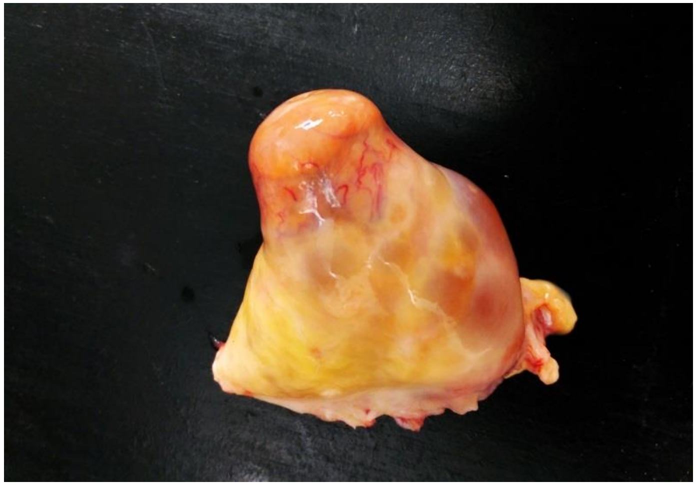
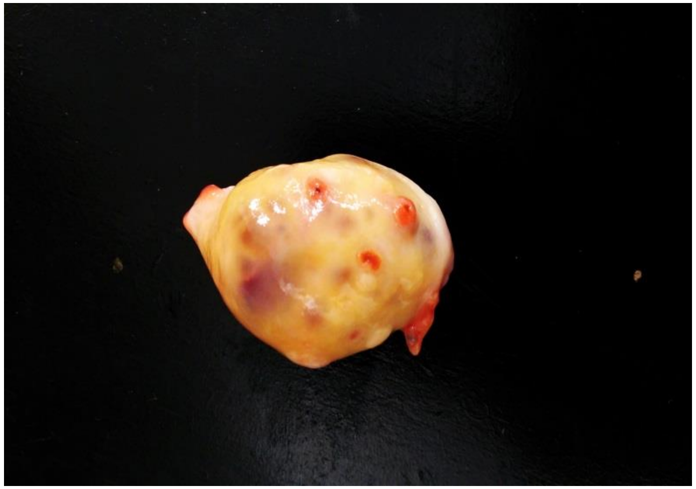
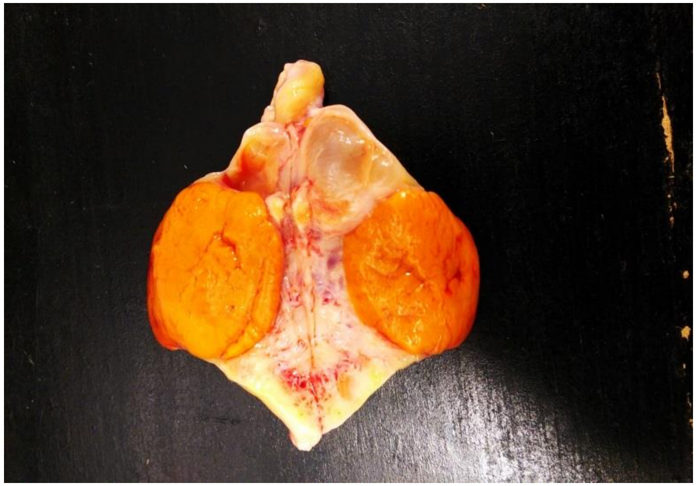
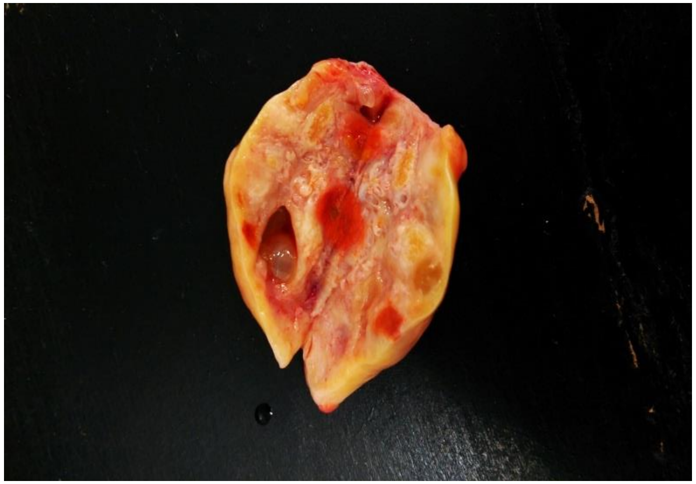

Tract ID LPS 4
08-07-15

**Left (used for experiment)**	**Right**
Ovary:<br>CL = medium/large<br>Follicles = 2 medium (~1.1cm) and few small<br>Size L/W/H (cm) = 4/2/1.8	Ovary:<br>CL = 3 old<br>Follicles = 1 medium and few small<br>Size L/W/H (cm) = 3.5/2/2
Horn:<br>Mucus = no<br>Caruncles = yes<br>Oedema = no<br>Colour = pink/normal	Horn:<br>Mucus = no<br>Caruncles = yes<br>Oedema = no<br>Colour = pink/normal
Oviduct:<br>vascularization = normal<br>Colour = pink	Oviduct:<br>vascularization = normal<br>Colour = pink

Cervix:
Mucus = small clear secretion
Open = no

Straw information:
- *National Cattle Breeding centre*
- Storage solution = white
- Straw ID = LEANAMORE OMAN IE191038420409

ID:LPS 4
08-07-15

LEFT

RIGHT

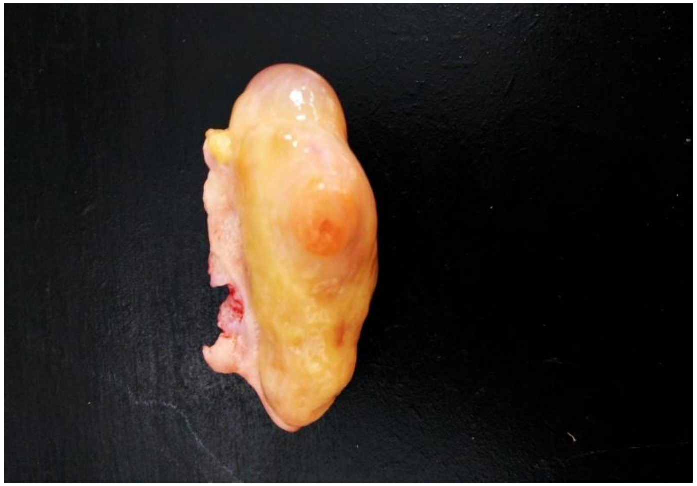

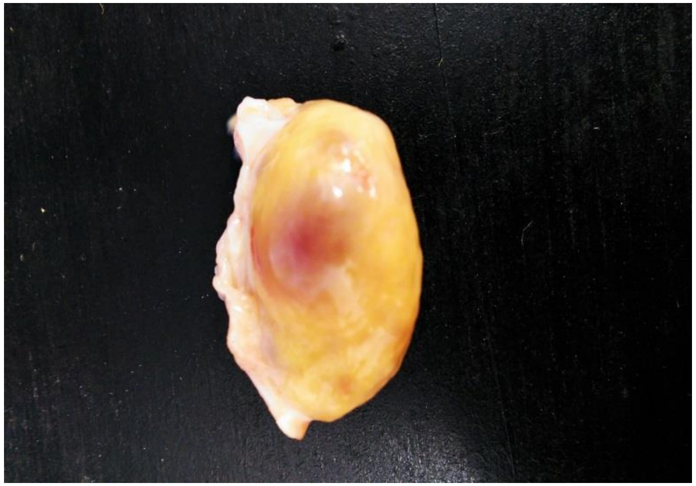

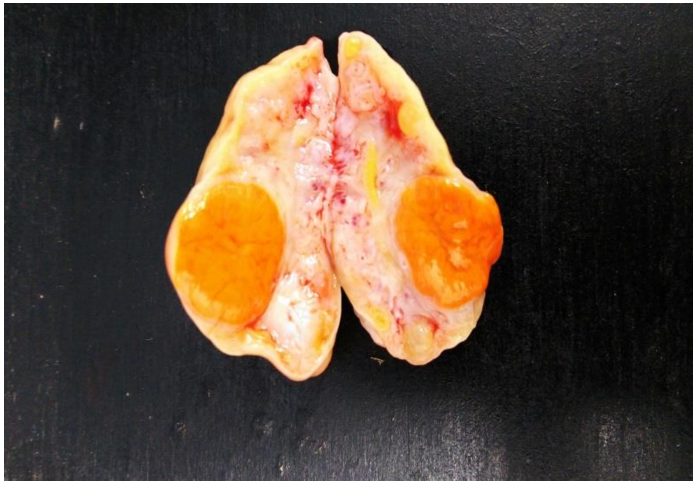

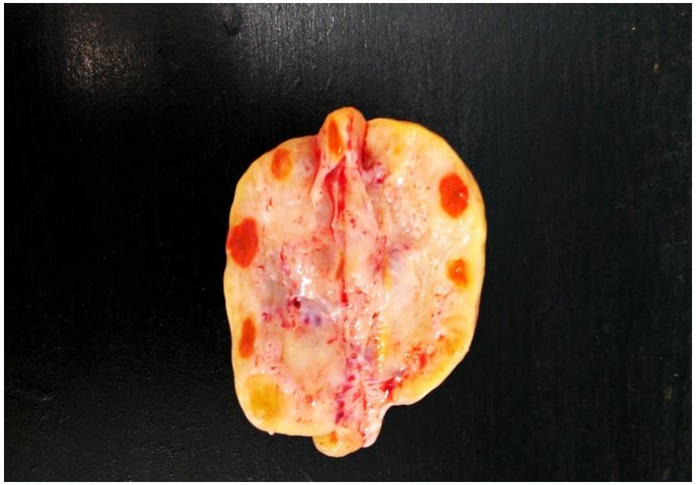

Tract ID *LPS* 5
08-09-15

Left	Right (used for experiment)
Ovary:<br>CL =<br>Follicles =<br>Size L/W/H (cm) = 3.5/2.8/2	Ovary:<br>CL = 1 large red/orange vascular CL and few old<br>Follicles = numerous small<br>Size L/W/H (cm) = 4.6/3/2.2
Horn:<br>Mucus = no<br>Caruncles = yes<br>Oedema = no<br>Colour = pink/normal	Horn:<br>Mucus = no<br>Caruncles = yes<br>Oedema = no<br>Colour = pink/normal
Oviduct:<br>vascularization = swollen<br>Colour = white	Oviduct:<br>vascularization = no<br>Colour = pink/normal

Cervix:
Mucus = no
Open = no

Straw information:
- *National Cattle Breeding centre*
- Storage solution = white
- Straw ID = LEANAMORE OMAN IE191038420409

ID:LPS 5
08-09-15

LEFT

RIGHT

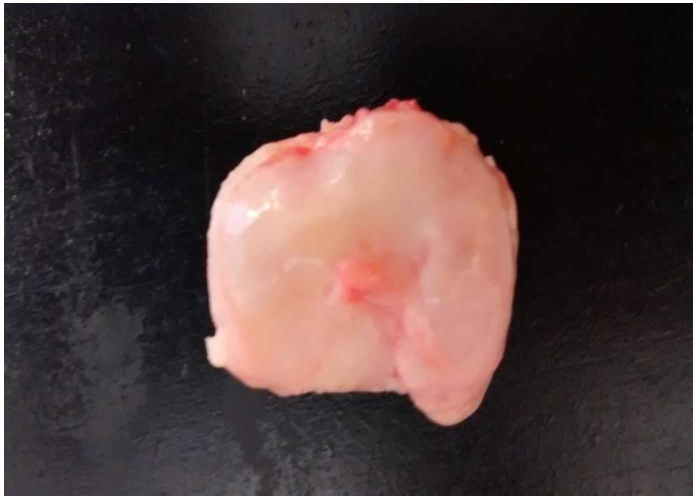

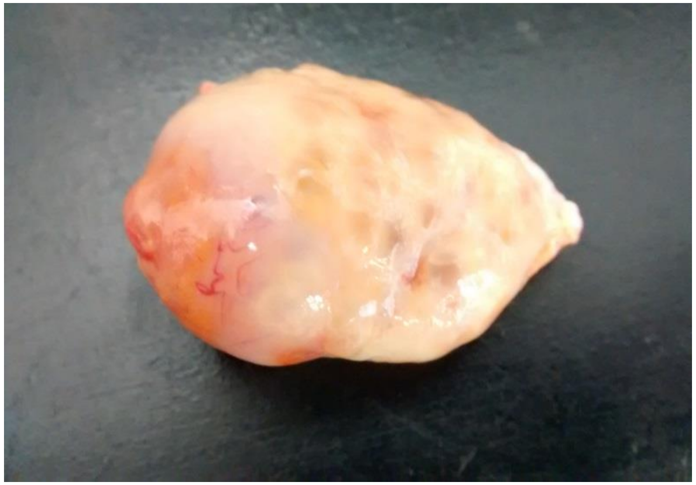

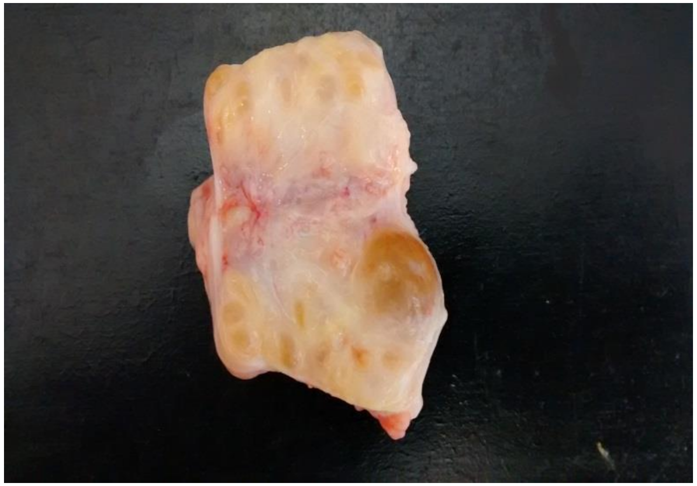

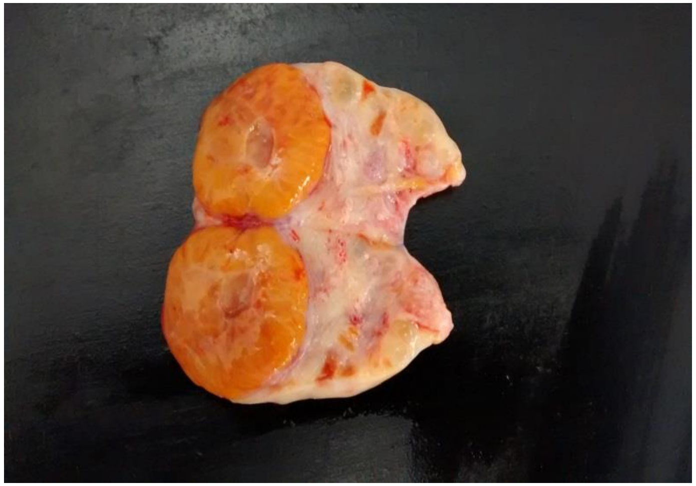

Video Legends

Videos 1 and 2 - Representative videos of sperm motility with and without *LPS*-challenge. Video 1 is an example of sperm bound to the ampulla epithelium under control conditions. Video 2 is a similar Example of sperm under *LPS* conditions.

Videos 3 and 4 - Representative videos of transport of particles in the ampulla with and without *LPS*-challenge. Video 3 is an example of particle transport in the ampulla under control conditions. Video 4 is a similar example of particle movement under *LPS* conditions.

Videos 7 and 8 - Representative videos of cilia beating with and without *LPS*-challenge. Video 5 is an example of ciliated cells of the ampulla epithelium under control conditions. Video 6 is a similar example of ciliated cells under *LPS* conditions.
